# Surfactant Administration Through Laryngeal or Supraglottic Airways (SALSA): A Viable Method for Low-Income and Middle-Income Countries

**DOI:** 10.3389/fped.2022.853831

**Published:** 2022-03-16

**Authors:** Henry A. Zapata, Prem Fort, Kari D. Roberts, Dinushan C. Kaluarachchi, Scott O. Guthrie

**Affiliations:** ^1^Department of Pediatrics, Division of Neonatology, University of Wisconsin-Madison School of Medicine and Public Health, Madison, WI, United States; ^2^Department of Pediatrics, Maternal, Fetal, Neonatal Institute, Johns Hopkins All Children's Hospital, St. Petersburg, FL, United States; ^3^Department of Pediatrics, Division of Neonatology, Johns Hopkins University School of Medicine, Baltimore, MD, United States; ^4^Department of Pediatrics, Division of Neonatology, University of Minnesota, Minneapolis, MN, United States; ^5^Department of Pediatrics, Division of Neonatology, Vanderbilt University School of Medicine, Nashville, TN, United States

**Keywords:** surfactant, RDS, LISA, LMA, SALSA, global health, low- and middle-income countries (LMIC), aerosolized surfactant

## Abstract

Administration of liquid surfactant through an endotracheal tube for the treatment of respiratory distress syndrome has been the standard of care for decades. A skilled health care provider is needed to perform this procedure. In lower-income and middle-income countries (LMICs), healthcare resources are often limited, leading to increased mortality of premature infants, many of whom would benefit from surfactant administration. Therefore, having a simplified procedure for delivery of surfactant without the need for advanced skills could be life-saving, potentially diminish gaps in care, and help ensure more equitable global neonatal survival rates. Modifications to the standard approach of surfactant administration have been put into practice and these include: INtubation-SURfactant-Extubation (INSURE), thin catheter surfactant administration (TCA), aerosolized surfactant, and surfactant administration through laryngeal or supraglottic airways (SALSA). Although there is a need for larger studies to evaluate the comparative effectiveness of these newer methods, these methods are being embraced by the global community and being implemented in various settings throughout the world. Because the SALSA technique does not require laryngoscopy, a provider skilled in laryngoscopy is not required for the procedure. Therefore, because of the ease of use and safety profile, the SALSA technique should be strongly considered as a viable method of delivering surfactant in LMICs.

## Introduction

Respiratory distress syndrome (RDS) is one of the most frequently diagnosed diseases in preterm infants in the neonatal intensive care unit (NICU) (1). Exogenous surfactant therapy can be life-saving for newborn infants with RDS. Invasive methods, with airway manipulation by laryngoscopy and placement of an endotracheal tube, have been performed for decades and were the method for surfactant administration in the initial surfactant trials (2–4). Less invasive methods, such as INtubation-SURfactant-Extubation (INSURE) (5, 6), thin catheter administration (TCA) [represented by the minimally invasive surfactant therapy (MIST) and less invasive surfactant administration (LISA) techniques] (6–10), aerosolized surfactant, and more recently, surfactant administration through laryngeal or supraglottic airways (SALSA) have become available. Randomized controlled trials and systematic reviews have shown safety and efficacy of these newer methods (10–24).

While INSURE requires endotracheal intubation, the TCA techniques are considered less traumatic to the airway than the traditional invasive methods of surfactant administration. These techniques still require operator expertise as laryngoscope insertion is needed (25). Aerosolized surfactant does not require airway manipulation and is therefore, the least invasive of the newer techniques. An aerosolization device, however, is not yet commercially available, but a device is currently under review by the United States Food and Drug Administration.

The SALSA technique involves placement of a laryngeal mask or supraglottic airway into the posterior pharynx without the need for laryngoscopy. Liquid surfactant is then administered through the device (11). Surfactant can then be delivered in a variety of settings and skill levels through the ease and efficiency of this method.

In this manuscript we will review the pathophysiology of RDS and the impact that treatments such as surfactant and continuous positive airway pressure (CPAP) have had on the disease. The evidence, safety, efficacy, and outcomes for each of the newer methods of surfactant administration will also be reviewed and we will draw conclusions as to the applicability of these methods in the LMIC setting. We will conclude by providing instructions on how to perform the SALSA technique as it has unique and specific characteristics that make it ideal for the LMICs setting.

## Respiratory Distress Syndrome: Pathophysiology and Surfactant

RDS is the leading cause of respiratory disease in preterm infants (1, 26). The premature lung has a tendency for collapse at the alveolar level, where the surface tension of a primary alveolus will eliminate its air into a secondary alveolus, causing collapse of the primary alveolus while over-distending the secondary alveolus (27). Surfactant acts to decrease surface tension and maintain a balance between alveoli. This decreases the ventilation-to-perfusion mismatches which contribute to hypoxemia (28).

Preterm infants produce limited amounts of surfactant and commonly require exogenous instillation of surfactant to decrease their oxygen requirement and ameliorate the symptoms of RDS. This is why surfactant therapy can be life-saving (29). Following the initial surfactant studies by Fujiwara in 1980 (30), multiple clinical studies have shown that surfactant therapy dramatically improves survival and respiratory outcomes of preterm infants with RDS (31). This subsequently led to governmental approval of surfactant in high-income countries (HICs) and its addition to the World Health Organization's (WHO) essential medications list (32). As surfactant is becoming more readily available in LMICs, having a method of administering surfactant which requires minimal provider skill may have a significant impact toward achieving the goal of improving infant mortality in LMICs.

## Impact of Surfactant on Mortality and Future Directions

In the pre-surfactant era, the mortality rate of neonates with RDS was approximately 40% (1, 2). Since surfactant became commercially available in the 1990s, overall infant mortality decreased by 25% and mortality due to RDS decreased 56% based on a 9-year retrospective review from 1987 to 1995 (33). A United States retrospective cohort study by Ramanathan et al. of 14,173 preterm infants with RDS from 2005 to 2009, who were treated with three different types of surfactants, showed the all-cause-in-hospital mortality rates to range from 3.61 to 5.95% (34). In comparison, a prospective study from Karachi, Pakistan in 1997 showed the overall mortality for newborns with RDS was 81/200 (41%) with an increase up to 70% for newborns with birth weight <1,000 g (35). Another study examined 205 Pakistani neonates from 2009 to 2010 and showed an overall mortality rate of 33% and RDS mortality rate of 23% (36). Differences in rural vs. urban residence have also been found with higher mortality among premature infants of rural residence in Australia (aOR 1.26, 95% CI 1.07-1.48, *p* = 0.005) (37). Similar trends are seen in neonates born in community hospitals of Uganda with nearly twice the mortality rate as those infants born in a tertiary center in that country (33 vs. 17%) (38, 39).

While advances in therapies and available approaches in treatment of RDS have led to improved morbidity and mortality in premature infants, there is a lag to disseminate such technology from HICs to LMICs (40, 41). The NICU panorama is especially discouraging in Sub-Saharan countries as neonatal mortality rate (NMR) there is the highest in the world (38). The burden has remained high in Sub-Saharan countries even after implementation of the Millennium Development Goals in 2000 (MDGs) as children from those countries are 10 times more likely to die in the first month of life than a child born in HICs (38–42). For instance, NMR in 2019 from Ethiopia, Afghanistan, Pakistan, and overall LMICs to name a few was reported as 28, 36, 41, and 19 per 100,000 live births, respectively. In contrast, HICs reported 3 per 100,000 live births. United States, United Kingdom, Sweden, Norway, and Japan reported 4, 3, 1, 1, and 1 per 100,000 live births, respectively (43). Despite these dramatic differences, some countries have decreased their NMR in the last decades. For example, Peru reduced its NMR by 51% from 2000 to 2013. In doing so, they were able to meet the fourth MDG in reducing the under-five mortality rate by emphasizing effective and simple strategies that could be easily implemented (44).

In an effort to continue improving care and reducing infant mortality in LMICs, the WHO has recommended Sustainable Development Goals (SDG). This collection of 17 goals is designed to achieve a more sustainable future for all by 2030. One aim is for every country to reduce NMR to at least as low as 12 per 1,000 live births and end preventable deaths of newborns and children under 5 years of age (42). Thanks to this, there has been an increasing focus on early neonatal care and the implementation of treatments and practices ideal for the LMIC setting.

## CPAP for RDS Management

Continuous positive airway pressure (CPAP) is a simple, cost efficient, non-invasive respiratory support considered an optimal first line therapy for RDS. It aims to support functional residual capacity and prevent the need for mechanical ventilation (MV) (45, 46). CPAP alone without need for surfactant administration is an appropriate initial management strategy for mild RDS, but extremely premature infants may require subsequent surfactant administration (47). Numerous studies have tried to avoid intubation in the delivery room or prevent prolonged MV, known to cause lung injury, by administering early CPAP in very preterm infants (48). Currently, CPAP is used immediately after birth to stabilize the lower airways and prevent collapse of alveoli. The SUrfactant Positive Pressure and Oxygen Randomized Trial (SUPPORT) (49), a pivotal study by the National institute of Child Health and Human Development (NICHD), showed that premature infants indeed could be placed on CPAP support without the need for intubation and surfactant administration with similar outcomes between CPAP and surfactant groups. BPD or death rates were 48% in the CPAP group vs. 51% in the intubation group.

Despite these studies coming from HICs, CPAP should be implemented in every LMIC seeking to decrease their NMR as it is an effective treatment for RDS. This can be supported by a systematic review which evaluated the feasibility and efficacy of CPAP in LMICs. Thukral et al. showed that implementing a CPAP program could reduce in-hospital mortality by up to 66% along with a decreased need for MV in preterm infants (50). Because of this, CPAP should be of primary importance in treating any infant in LMICs with RDS. As LMICs continue to invest in reducing their NMR and surfactant use increases, easy and efficient ways to deliver surfactant will be needed.

## Exogenous Surfactant *via* ETT Administration

When there is a need to administer surfactant, the traditional approach has been to deliver exogenous surfactant through an endotracheal tube (ETT). However, less invasive methods, which may be considered “off label” use based on the country, are now widely being used around the world (11).

Neonatal endotracheal intubation is a life-saving and critical skill, but there are risks associated with it and this needs to be done by a neonatologist or health care provider with expertise in the procedure (51). This skill requires advanced training as well as continuous performance to maintain a level of competency deemed suitable for successful intubations (52, 53), thereby Leach relates “skill acquisition as a developmental process” that evolves over time (54, 55).

In areas without advanced neonatal care, a skilled health care provider capable of successful intubation may not be present in an urgent and timely manner when surfactant is needed in a critically ill infant. In these settings, less invasive methods could be an alternative.

## Insure Administration

INSURE is the most studied mode of surfactant instillation. The prompt removal of an ETT after instillation of exogenous surfactant was both feasible, safe, and beneficial to the premature infant (56). INSURE was developed in response to concerns that prolonged MV increased the risk of lung injury. In theory, this “short-time” intubation decreases the risk of barotrauma from MV, which can lead to lung injury, BPD, and death (6, 57).

INSURE is used to treat an infant who is on CPAP. An ETT is inserted, and exogenous surfactant is then instilled *via* the ETT (5). The infant is not placed on MV as the ETT is removed immediately. Isayama et al. showed a decrease in both BPD and death when INSURE was used and the infant was not exposed to MV (OR, 0.71; 95% CI, 0.50-0.98) (56).

INSURE, however, requires a skilled provider as endotracheal intubation is still required. In a recent systematic review by De Bisschop et al. of 1,674 patients, one third (median of 33%) of infants failed and required MV after using INSURE (5). This technique is widely practiced clinically for infants with low oxygen requirements, who may benefit from surfactant administration without the need for continued MV. To our knowledge, and due to still needing expertise, no studies have demonstrated the effectiveness of using INSURE in LMICs.

## Thin Catheter Administration

Several terms are used to describe the variations of the TCA method. The most common are: Less Invasive Surfactant Administration (LISA), Minimally Invasive Surfactant Therapy (MIST), Take Care, and SurE (58). TCA involves the delivery of exogenous surfactant by means of a thin catheter such as a feeding tube or angio-catheter. This is inserted into the trachea of a spontaneously breathing infant on CPAP. The premise behind this method involves avoidance of brief intubation as with the INSURE technique and the avoidance of any positive pressure breaths which may cause alveolar damage (59). The ultimate goal is to reduce complications due to airway manipulation and improve the success of CPAP after surfactant administration (6).

A recent metanalysis that compared methods of non-invasive surfactant administration concluded that surfactant administration *via* thin catheters was associated with lower likelihood of mortality, need for mechanical ventilation and bronchopulmonary dysplasia compared with InSurE (60). Like INSURE, performance success with TCA is dependent on a skilled provider as the skillset is nearly identical to intubation, but involves a thin and more delicate tube and possibly the use of Magill forceps. Systematic reviews and meta-analysis showed that LISA was associated with reduced composite outcome of death or BPD at 36 weeks compared with standard intubation (58, 60, 61). Most recently, the OPTIMIST-A trial assessed the effectiveness of surfactant administration *via* MIST in extremely preterm infants with RDS managed on CPAP. The overall composite BPD or death was not significantly reduced in the TCA group compared with CPAP alone (43.6 vs. 49.6%), while death was not significantly different (10 vs. 7.8%), BPD at 36 weeks' postmenstrual age was lower in the MIST group (37.3 vs. 45.3%) (62).

Jena et al. of India, have shown that TCA administration can be applicable to an LMIC setting. They found that, compared with the INSURE group, TCA infants had less need for subsequent MV (19 vs. 40%), shorter duration of supplemental oxygen, shorter hospital stay, and a lower rate of supplemental oxygen at 36 weeks' corrected gestational age (63).

The TCA method may be feasible in an LMIC setting, but requires a skilled provider to use a laryngoscope to pass a catheter through the vocal cords. Thus, its applicability is primarily within a NICU environment.

## Aerosolized Surfactant Administration

One of the most recent developments in surfactant therapy has been aerosolized or nebulized surfactant. With this method, surfactant is administered by means of a delivery device which nebulizes or aerosolizes the drug, allowing it to be inhaled and then distributed to the airways eventually reaching the alveoli. Currently, there are few trials that reveal promising benefits toward the use of aerosolized surfactant (24, 64–66). Minocchieri et al. showed reduction in the need for intubation in infants who were randomized to receive nebulized surfactant (RR 0.526, 95% CI 0.292-0.950) in infants born at 29-33 weeks gestational age (66). The largest multicenter randomized control trial done by Cummings et al. performed on 457 infants between 23 and 41 weeks' gestation with median 33 weeks of GA showed a decreased need for intubation by nearly 50% (50 % in usual group vs. 26% in aerosol group) in an intent-to-treat analysis (*p* < 0.0001; RR: 0.51 90% CI: 0.41-0.63) (24). In a systematic review of ~1,095 infants, the aerosolized surfactant group had a significantly reduced intubation rate compared with the standard care group at 72 h after birth. The intubation rate was lower in infants > 28 weeks' gestation with no differences in neonatal morbidities or mortality identified (67).

An advantage of aerosolized/nebulized surfactant is that it is the least invasive method to deliver surfactant and requires minimal skills to administer. However, as of the writing of this review, there is currently not a device that is commercially available, although a device is currently under review by the United States Food and Drug Administration. If a device were available, this method will require specific equipment that may be difficult or cost-prohibitive to obtain in LMICs, thereby limiting its application in this setting.

## Salsa

**Figure 1:**
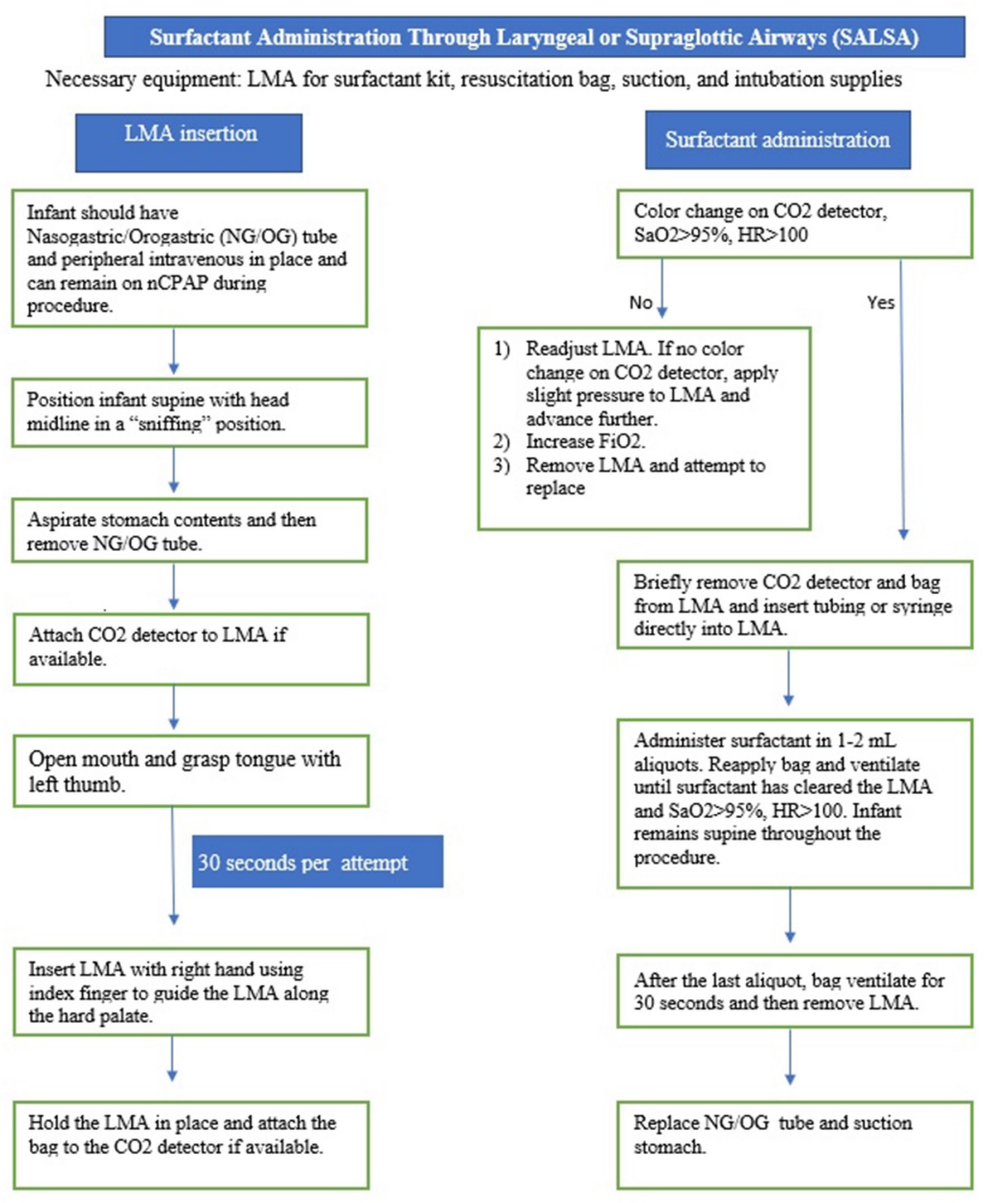
SALSA: Step-by-step guidelines.

**Figure 2:**
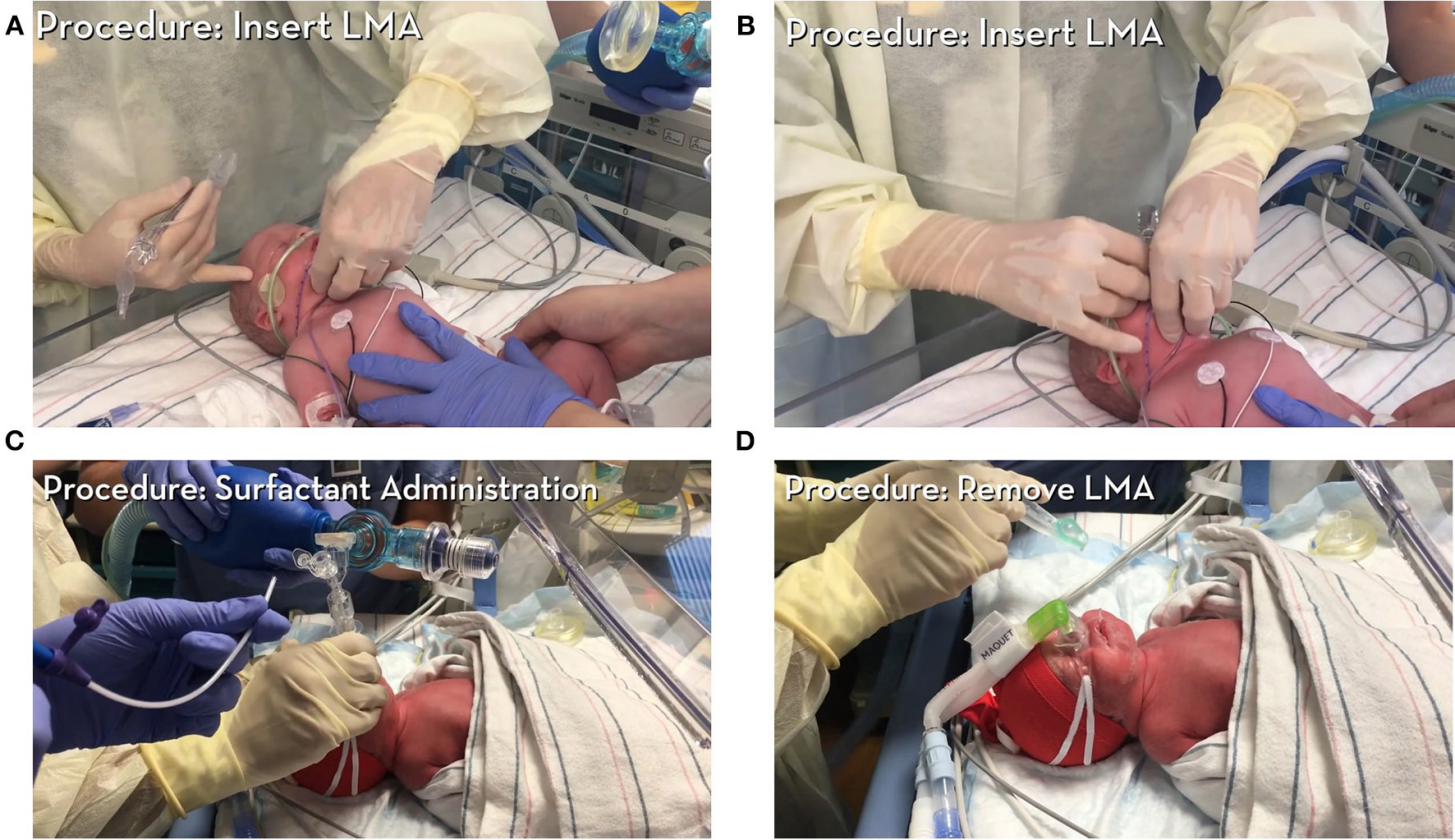
Instructional video 1 provides an overview and step-by-step instructions on how to administer surfactant *via* SALSA technique. All patient consents were obtained in accordance with policies at University of Minnesota (Courtesy of Dr. Kari Roberts). **(A)** Demonstrates insertion of LMA, **(B)** Illustrates final position of LMA, **(C)** A piece of tubing connected to the surfactant syringe is placed into the lumen of the device to deliver surfactant, **(D)** LMA removal.

**Figure 3:**
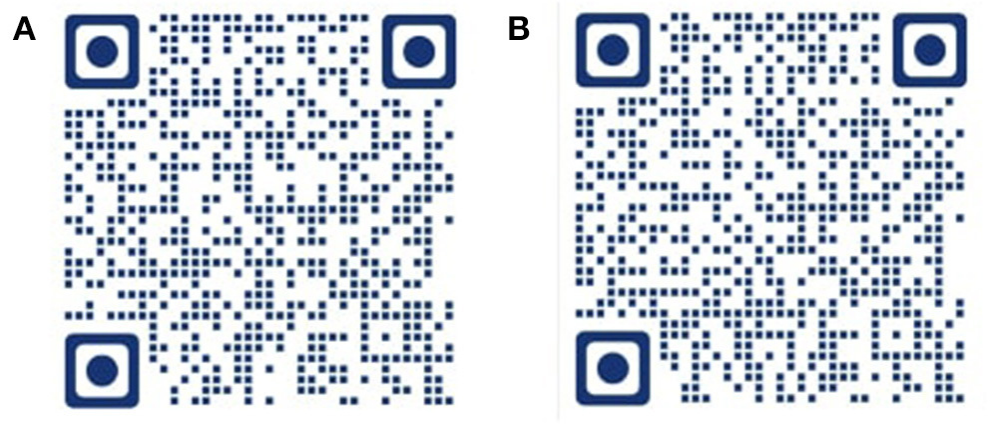
Access videos using the following QR codes. **(A)** access video 1, **(B)** access video 2.

SALSA is a less invasive method that has unique characteristics that makes it an ideal candidate for the LMIC setting. The SALSA technique consists of inserting a supraglottic airway device (SAD), commonly known by the trade name, Laryngeal Mask Airway (LMA), into the mouth and advancing it until the device is unable to be advanced further. Proper placement is indicated by listening for bilateral breath sounds and, if available, color change on a carbon dioxide detector. A piece of tubing connected to the surfactant syringe is then placed into the lumen of the device to deliver the surfactant in aliquots just above the vocal cords (11–23). Figures 1–3 provide a guide on how to perform the SALSA technique. Figure 1 provides a detailed step-by-step algorithm appropriate for the LMIC setting.

The SALSA technique differs from traditional intubation, INSURE, and TCA in that there is no insertion of the device through the vocal cords, therefore, use of a laryngoscope is not required for proper placement of the device. In addition, insertion of the LMA is substantially easier and faster than the insertion of an ETT (68), and decreases the risk of trauma to the glottic and subglottic tissues.

In the largest randomized controlled trial of SALSA, placement of the device took an average of 88 s (range 12-500 s) and 67% of attempts were successful in <35 s (23). The device is simple and since it is placed without use of a laryngoscope, placement does not require a skilled health care provider (68). This is often ideal for an LMIC setting. In addition, studies have shown that the SALSA technique decreases the need for MV (14, 19, 21). When SALSA was compared to CPAP alone or surfactant administration by INSURE, there was a substantial decrease in the need for intubation and MV (RR 0.43; 95% CI 0.31-0.61) (12). Roberts et al. reported a significantly decreased rate of intubation and MV (38 vs. 64%) compared with control with a more rapid improvement in fraction of inspired oxygen (FiO2) (14). Similarly, Attridge et al. reported significant and persistent lower FiO2 in the LMA or SALSA group (69). SALSA has been shown to be safe, with no difference in adverse events and no significant increase in air leak, BPD, intraventricular hemorrhage, or mortality between SALSA and either CPAP alone or in combination with endotracheal intubation (70, 71).

With decreased need for MV, the SALSA technique may be beneficial for survival of infants in LMICs where access to MV is limited or not available. Because of this, SALSA was introduced in 2018 and has been widely used by physicians in the Republic of Azerbaijan. This has been one contributing factor to their recognition by WHO as one of the fastest progressors globally in meeting the SDG 2030 goals in reduction of the country's NMR (72, 73).

## Minimally Invasive Methods in Low-Income and Middle-Income Countries With Limited or no Advanced Neonatal Care Available

According to the WHO, nearly 15 million babies are born prematurely each year. Preterm birth complications are considered the leading cause of death among children under 5 years of age. This accounted for 1 million deaths in 2015 (74). United Nations Children's Fund (UNICEF) data showed that globally 2.4 million children died in the first month of life in 2020 (75). Countries' emphasis in improving NMR are making a difference as 5 (4.9-5.2) million died in 1990 (76). In most regions around the world, deliveries are not attended by a physician or skilled health care provider. A large study of term and preterm infants (*n* = 612) in Ethiopia found that only 39% of attendants at deliveries were medical doctors or general practitioners and only half (53%) of the births occurred at a referral hospital (77).

Studies in adult and pediatric patients show that intubation at referring or community settings, where there is less access to a skilled airway management, increases the risk of poor outcomes, including death (53, 78, 79). Recognizing this, the International Liaison Committee on Resuscitation (ILCOR) recommended the LMA device in neonatal resuscitation because of its availability and ease of use and when intubation is not feasible (18, 80). This was subsequently adopted by the Neonatal Resuscitation Program and the European Resuscitation Council (81, 82).

INSURE and TCA methods for administration of surfactant do show a decrease in the need for MV. Because of this, these methods have been implemented as standard practice in many parts of the world. The need for a high degree of skill which is similar to intubation does make this method less desirable for areas where advanced neonatal care and the equipment required to provide this care is not available. Inhaled surfactant, although promising, still requires an approved device to determine whether it will be a widely accepted method, but the cost and additional resources required will likely not allow this method to be widely used outside of high resource settings.

SALSA requires minimal skill and resources and is feasible in areas where advanced neonatal care is not widely available (14). This is seen by low pediatrician density in LMICs, which produces a high burden and inequities of distribution of healthcare provider (83).

Although we recognize the limitations of small, unblinded, clinical trials, and that larger trials are necessary to establish a complete safety and efficacy profile, SALSA should become a preferred alternative method to administer surfactant in infants that require this life-saving medication in the LMICs setting. Furthermore, it is ideal for use in situations where there is no provider skilled at intubation, CPAP is available, and/or a minimally invasive method of surfactant administration is desired. If health care providers are trained to administer surfactant using the SALSA technique, it may show benefits in reduction of oxygen needs, intubation and MV, but would also be expected to improve overall morbidity and mortality in newborns with RDS.

Unlike intubation which requires significant training to acquire skills, placement of a SAD can be learned quickly with a higher successful rate at first attempt placement (23). To assist with education and dissemination of the SALSA technique, a treatment protocol appropriate for the LMIC setting is provided in this review. Links to educational resources available on the internet to train a provider in this method have also been made available.

Detailed instructions on how to perform the SALSA technique are available in the following links: https://www.youtube.com/watch?v=Iig9l4BgIy4 and https://www.youtube.com/watch?v=ioXGyfVLdyE.

Figure 3 shows QR codes that can be scanned with a phone to facilitate access to these videos. These videos should familiarize clinicians with the equipment and step-by-step instructions so they feel comfortable performing the procedure in the clinical setting. In our experience, this is frequently the only training a LMIC provider needs in order to perform the procedure correctly.

## Limitations and Future Directions

The benefits of surfactant are clear. The methods to deliver surfactant will, however, continue to evolve. Each of the techniques surveyed in this review have demonstrated safety and efficacy. It is the opinion of the authors that the SALSA technique is uniquely applicable to the LMICs setting. Establishing programs to implement the SALSA technique in LMICs may be advantageous to improve NMR in countries that lack skilled providers but have access to surfactant. One such initiative took place in Azerbaijan and was backed by the Ministry of Health (72). Other countries where this method has been taught and implemented include: Tanzania, Jordan, and Brazil among others. Further studies are needed to evaluate neonatal outcomes post-treatment.

Similarly, the SALSA technique could be applied to community hospitals in HICs to help improve outcomes. This may include the prevention of transfers to tertiary care facilities, thus decreasing costs and health care utilization. Quality-improvement programs with stepped-wedge design could give more insight into the challenges of implementation across different hospital systems and how this could be improved. Leveraging social media's power to spread medical science and literature may also be of great benefit to shorten the time it takes to disseminate and implement evidence-based therapies from HICs to LMICs.

Finally, it is recognized that at this time SADs are not widely available in most LMICs and that providers may not have experience with the device. As surfactant use becomes more widespread, an investment in education and a dissemination of the tools needed to deliver this medication is equally important. Another limitation is that currently available SADs are too large for infants weighing < ~1,200 g. Development of a cost-effective SAD designed to administer surfactant over a broad range of infant weights would benefit LMICs wishing to use surfactant in combination with CPAP to improve their infant mortality rates. When this occurs, it should be evaluated not only in a HIC setting, but in LMICs where we expect the greatest benefit will be found.

## Author Contributions

SG, PF, and HZ: conception and design. HZ, PF, KR, DK, and SG: draft manuscript preparation. All authors reviewed and approved the final version of the manuscript.

## Conflict of Interest

PF, KR, and SG have worked for and received payments from ONY Biotech in the past. The remaining authors declare that the research was conducted in the absence of any commercial or financial relationships that could be construed as a potential conflict of interest. The reviewer AP declared past co-authorships with one of the authors KR and the absence of any ongoing collaboration with any of the authors to the handling editor.

## Publisher's Note

All claims expressed in this article are solely those of the authors and do not necessarily represent those of their affiliated organizations, or those of the publisher, the editors and the reviewers. Any product that may be evaluated in this article, or claim that may be made by its manufacturer, is not guaranteed or endorsed by the publisher.
